# Tug of war: innate immunity and herpes simplex keratitis

**DOI:** 10.3389/fimmu.2025.1658579

**Published:** 2025-08-29

**Authors:** Preston Nguyen, Betty Jacobs, Athul Mohanram, Caleb Hammons, Junji Xing

**Affiliations:** ^1^ Immunobiology and Transplant Science Center, Department of Surgery, Houston Methodist Academic Institute, Houston Methodist, Houston, TX, United States; ^2^ Department of Cardiovascular Sciences, Houston Methodist Academic Institute, Houston Methodist, Houston, TX, United States; ^3^ Department of Surgery, Weill Cornell Medicine, Cornell University, New York, NY, United States

**Keywords:** herpes simplex keratitis (HSK), herpes simplex virus type I (HSV-1), innate immunity, DNA sensor, therapeutic treatment

## Abstract

Herpes simplex keratitis (HSK), caused by herpes simplex virus type I (HSV-1) ocular infection, is a leading cause of visual morbidity worldwide, and although cases of HSK can be managed with current medications, new developments are required to make treatments more effective and satisfactory. Current evidence suggests that corneal scarring and vascularization result from chronic inflammation triggered by HSV-1 antigens. The pathogenesis of HSK remains complex and incompletely understood, but there have been many recent advancements have improved our knowledge of HSV-1 and its interactions with the host immune system, particularly in regard to various signaling pathways and regulators. In this review, we discuss the roles of innate immunity in corneal epithelial cells and innate immune cells, DNA sensors and regulators of DNA sensing pathways in HSK caused by acute and recurrent HSV-1 ocular infection and present potential immune-based therapeutic targets for novel HSK treatments.

## 1 Introduction

Herpes simplex keratitis (HSK) is a leading cause of infectious blindness worldwide (1, 2). HSK is primarily caused through ocular infection by herpes simplex virus type 1 (HSV-1), a member of the Herpesviridae family commonly associated with oral infections (3). Based on data from 2020, the global incidence of HSK is estimated to be 24 cases per 100,000 people, translating to around 1.7 million cases per year (4). HSV-1 is a double-stranded DNA (dsDNA) virus that exhibits a strong neurotropic nature, meaning that it infects and persists in neuronal tissues (3, 5). After primary infection through mucosal or skin contact, HSV-1 travels retrograde along sensory nerve axons to establish latent infection within the trigeminal ganglia (6). During the latent phase, HSV-1 produces latency-associated transcripts (LATs), which maintain the virus’s non-replicative state and prevent host immune clearance (7). HSV-1 can then periodically reactivate, resuming viral replication in response to various stimuli such as stress, fever, ultraviolet (UV) exposure, or immunosuppression (3). The reactivated virus travels anterogradely to the cornea, causing recurrent HSK, which can be split into several clinical subtypes, such as stromal, epithelial, and endothelial, based on the affected corneal layer (1, 8). A study on tree shrews has shown that the HSV-1 viral genes are still active in the corneal and ciliary ganglion tissues even after the acute infection, which demonstrates the complex pathogenesis of this virus since it can have multiple reservoirs (9). Other experimental models are widely used to study HSV-1 keratitis as well, including murine models (10–13), which have been fundamental in dissecting innate and adaptive mechanisms (14); rabbit models (15), which has been used to study corneal latency; and guinea pig models (16), which have provided insights into ocular viral shedding. Because of the high rate of recurrence, several complications may occur, including ulcerations, scarring, and blindness (17). Blindness mainly results from an exaggerated inflammatory response by innate immune cells to HSV-1 infection (18). Innate immunity serves as the first line of defense against HSV-1 (19), playing a critical role in controlling HSK. Understanding the mechanisms by which antiviral innate immunity regulates HSK and how HSV-1 evades these defenses in innate immune cells is essential. Pattern recognition receptors (PRRs), such as DNA sensor cyclic GMP-AMP synthase (cGAS) (20), on innate immune cells detect pathogen-associated molecular patterns (PAMPs) from HSV-1, such as dsDNA, triggering downstream DNA-sensing signaling pathways (19, 21). These pathways recruit innate immune cells, including dendritic cells (DCs), macrophages, natural killer (NK) cells, and neutrophils, to the infection site. These cells secrete inflammatory molecules, promoting effects such as enhanced cell metabolism and further immune cell recruitment (1, 22). Diagnosis of HSK is primarily clinical, and it is usually supplemented with a slit-lamp examination, which uses using a low-power microscope to provide a detailed view of the eye’s structures (8, 23). HSK is typically treated with the antiviral drug acyclovir, which is often supplemented with topical corticosteroids depending on the HSK subtype (24). Alternative approaches for treating HSV-1 include gene-editing strategies, such as mRNA-carrying lentiviral particles delivering SpCas9 mRNA and viral-gene-targeting guide RNAs. These methods have demonstrated inhibition of HSV-1 replication in preclinical studies (2). However, managing HSK remains challenging due to high recurrence rates, immune-mediated corneal damage, and impaired corneal nerve regeneration. Current treatments cannot prevent viral latency or reactivation (8, 25).

## 2 Classification and pathophysiology of HSK

According to the clinical signs of HSV-1 infection in the cornea, HSK is classified into different clinical types, including epithelial HSK, stromal HSK, and endothelial HSK. Their distinct pathological processes and immune mechanisms are discussed below.

### 2.1 Epithelial HSK

Epithelial HSK is the most common form of ocular HSV-1 infection and is characterized by active viral replication in the corneal epithelium, which is the outermost layer of the eye’s cornea that plays a vital role in vision and protection, resulting in the destruction of corneal epithelial cells (CECs) (18, 26). It presents as dendritic ulcers, which are superficial corneal ulcers that extend in tree-like patterns, and geographic ulcers, which are a progression of dendritic ulcers and appear as amoeboid-shaped ulcers with scalloped borders (27). The primary symptoms are eye pain, photophobia, tearing, decreased vision, and reduced corneal sensitivity (23). During reactivation, HSV-1 travels from the trigeminal ganglion via the ophthalmic nerve branch and infects corneal epithelial cells, resulting in localized inflammation and corneal scarring (28). PRRs detect dsDNA and other PAMPs from HSV-1, initiating a type I interferon (IFN) response and releasing inflammatory cytokines and chemokines (22, 29–31). The first responders in epithelial HSK are neutrophils, which clear the virus while also causing tissue damage through reactive oxygen species (ROS) (32).

### 2.2 Stromal HSK

Unlike epithelial HSK, which occurs due to active viral replication, stromal HSK is primarily immune-mediated, meaning that it can occur without detectable viral presence due to the immune system continuing to react even after the virus has been cleared. It is characterized by recurrent inflammation in the corneal stroma, which is the thickest layer of the cornea that provides structural support and facilitates wound healing, and its primary symptoms are scarring, thinning, and vision loss (18, 33). Stromal HSK is a CD4^+^ T-cell-mediated delayed-type hypersensitivity (DTH) reaction, meaning that even after the initial HSV-1 infection is resolved, CD4^+^ T cells become activated and secrete pro-inflammatory cytokines, recruiting and activating local macrophages that cause inflammation and tissue damage in the corneal stroma (1, 34).

### 2.3 Endothelial HSK

Endothelial HSK is characterized by inflammation of the corneal endothelium, the innermost layer of the cornea responsible for nutrient transport and maintaining corneal deturgescence, and it can lead to stromal edema, keratic precipitates, iritis, and elevated intraocular pressure (18, 35). Like stromal HSK, endothelial HSK is primarily immune-mediated and occurs due to a reactive hypersensitivity response to viral antigens in the corneal endothelium that persist even in the absence of live virus (36). Antigen-presenting cells (APCs), such as DCs and macrophages, can migrate to the cornea and express major histocompatibility complex class II (MHC-II) molecules, which activate CD4^+^ T cells (37). CD4^+^ T cells infiltrate the posterior stroma and endothelium and produce cytokines in response to residual HSV-1 antigens in the endothelium, activating resident immune cells and leading to the inflammation of the endothelium (38).

## 3 Role of CECs in HSK

**Figure 1:**
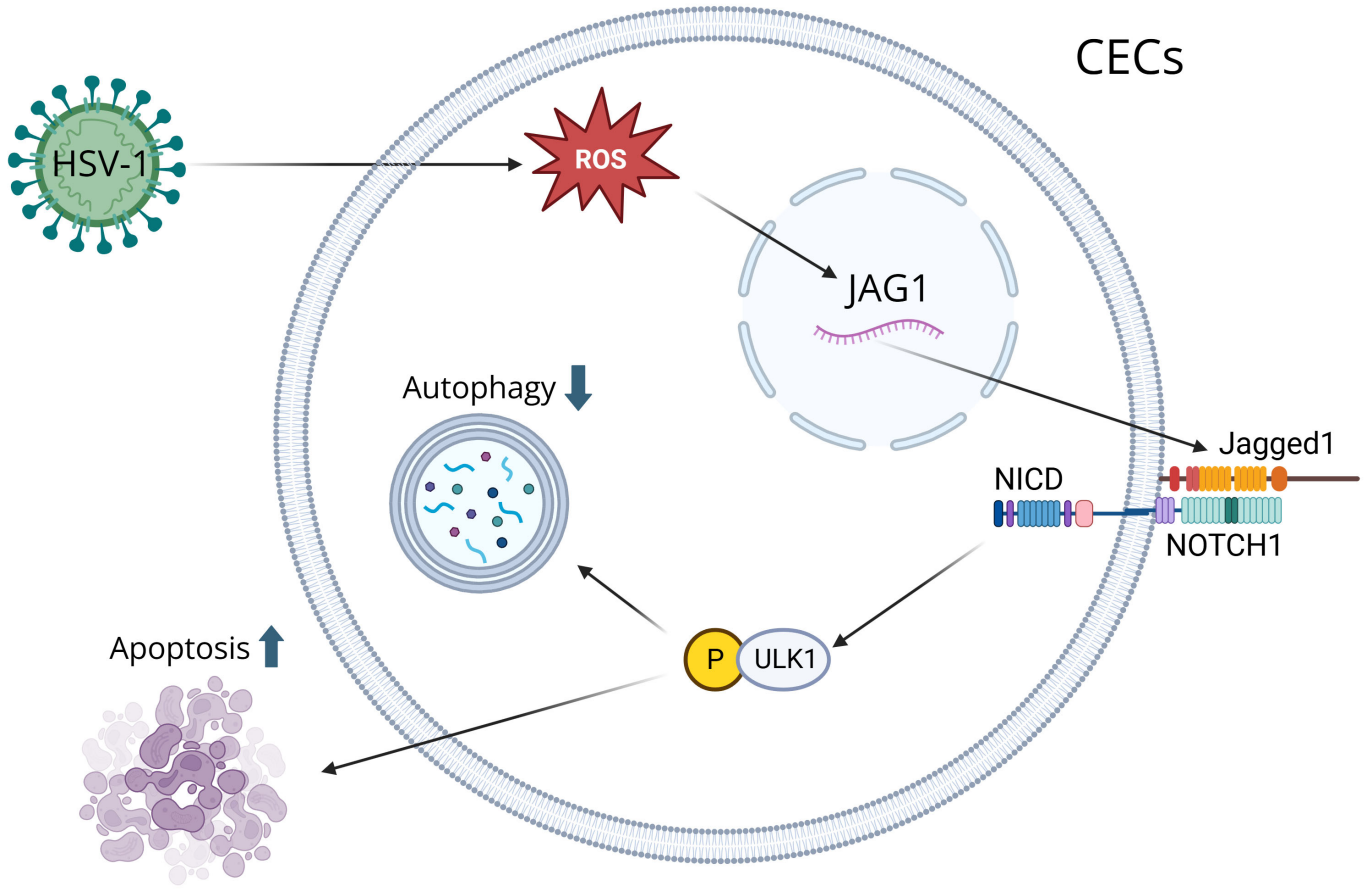
A visual diagram that illustrates how HSV-1 induces corneal epithelial cells (CECs) apoptosis by suppressing autophagy through the ROS-JAG1-NOTCH1-pULK1 signaling pathway. Reactive oxygen species (ROS) are produced during HSV-1 infection, inducing the Jagged1 (JAG1) signaling pathway. ROS modulates JAG1 expression, and when JAG1 binds to a notch receptor on a neighboring cell, it triggers a series of proteolytic cleavages to release the Notch Intracellular Domain (NICD). NICD interacts with ULK1 in the cytoplasm, resulting in its phosphorylation. Through mitochondrial interaction, ULK1 inhibits autophagy and leads to apoptosis.

CECs are the outermost layer of cells that cover the front surface of the cornea, and studies have shown that CECs secrete extracellular vesicles whenever the cornea is wounded (39). The immune response of the cornea is predominantly controlled by Anterior Chamber Immune Deviation (ACAID), which prevents the immune system from responding too extremely to various particles, microorganisms, or viruses that enter the eye, which protects the eye from inflammation that can lead to blindness (40). ACAID is initiated when APCs capture antigens in the anterior chamber and migrate to the spleen, where they induce the expansion of regulatory T cells (Tregs). These Tregs secrete immunosuppressive cytokines, such as interleukin-10 (IL-10), which suppress Th1-driven and Th17-driven responses that would otherwise promote neutrophil and macrophages infiltration and corneal scarring (41). By promoting the activity of CD4^+^ and CD8^+^ Tregs, ACAID reduces the risk of destructive stromal inflammation by suppressing antigen-specific DTH and effector T cell activity (42). The CECs play an important role in the immune response because they recognize PAMPs and damage-associated molecular patterns (DAMPs), activating neutrophils and causing inflammation (1). After HSV-1 infection, ROS are produced in CECs, which is essential for activating key immune signaling pathways (43). Increased ROS induces Jagged1 (JAG1) expression, and the JAG1-NOTCH1-pULK1 pathway inhibits autophagy and leads to apoptosis of CECs since increased JAG1 leads to the activation of pULK1, which suppresses autophagy and leads to apoptosis (44) (**Figure 1**). CECs also exhibit antiviral functions, notably through the production of type III IFN. CECs primarily produce type III IFN, which suppresses viral replication and modulates the inflammatory response, and they also produce type I IFN, which activates antiviral mechanisms and recruit immune cells to the site of the infection (45). CECs secrete extracellular vesicles carrying proteins, lipids, and signaling molecules upon injury, which activate neutrophils and initiate inflammation (46). They also produce cytokines, such as IL-18 and IFN-γ, to recruit DCs and macrophages, which process antigens and present them to T cells, activating the adaptive immune response (47).

## 4 Role of innate immune cells in HSK

### 4.1 DCs

DCs are a special type of antigen-presenting cell that act as the “sentinels” of the immune system and bridge the innate and adaptive immune system by presenting antigens to T cells (48–50). It used to be believed that there were no DCs directly on the cornea, but now it is known that there is a stratified network of DCs throughout the cornea (51). The interesting thing about DCs is that a study has shown that reducing DCs reduces the severity of stromal disease since DCs induce an inflammatory response through the activation of T cells, causing more damage (52). Tripartite Motif 29 (TRIM29) is strongly induced by cytosolic dsDNA in DCs, and TRIM29 deficiency has been shown to increase resistance to HSV-1 through increasing the production of type I IFN (23), suggesting the possible role of TRIM29 in controlling HSK. There are several subsets of DCs present in the peripheral cornea including CD11c^+^ conventional DCs (cDCs) and plasmacytoid DCs (pDCs). In murine HSV-1 infection, resident cDCs promote local recruitment of NK cells and inflammatory monocytes, which leads to early viral clearance (53). While cDCs promote systemic viral dissemination, resident pDCs play a protective role by limiting viral burden and preserving the function of Tregs, making them extremely important in preventing clinical disease and nerve loss (37). Before infection, many corneal DCs are in an immature state, which supports ACAID. This regulatory environment favors tolerogenic DCs that induce Tregs and dampen inflammation (54). However, upon HSV-1 infection corneal DCs undergo rapid maturation, upregulating MHC-II and producing pro-inflammatory cytokines, thereby promoting the differentiation of effector CD4^+^ T cells, which contribute to stromal immunopathology (52).

### 4.2 Macrophages

Macrophages are another type of white blood cell that remove dead cells, kill microorganisms, and stimulate other immune cells (55, 56). Unlike DCs, macrophages are not present in naive corneas, but CCR2^+^ migratory macrophages are the predominant innate infiltrate within 48 hours, contributing to early viral sensing and cytokine production (57). M1 macrophages are classically activated and produce pro-inflammatory mediators such as IL-6 and TNF-α. These responses promote viral clearance while also causing corneal damage through the recruitment of neutrophils and the amplification of stromal inflammation (58). In contrast, M2 macrophages are alternatively activated and secrete anti-inflammatory mediators like IL-10, which supports tissue repair, resolution of inflammation, and angiogenesis (59). A study has been done that tested a ganglioside GM1 liposome vaccine that encapsulated HSV-1 glycoprotein D and targeted CD169^+^ macrophages, and the study showed that the vaccine increased the number of corneal infiltrating macrophages, polarizing them toward M1, and there were also significantly more T cells and DCs (10). The Mal adaptor protein plays an important role in TLR9 signaling through ERK1/2 kinases, making it essential for TLR9-mediated expression of IFN-β and TNF-α in macrophages exposed to HSV-1 (60). Macrophages play a key role in the early immune response to HSV-1 in the olfactory epithelium, causing inflammation as the virus spreads from the apical layers to the basal layers and into the underlying tissues (61). Furthermore, the deletion of TRIM18 increases the production of type I IFN response in macrophages, protecting mice from HSV-1 infection (62), suggesting the possible role of TRIM18 in HSK. In addition, overexpression of NOD-like receptor family pyrin domain containing 12 (NLRP12) triggers IL-18-meidated pyroptosis in infected macrophages, amplifying antiviral signaling cascades to alleviate HSK (63).

### 4.3 Innate lymphoid cells

ILCs are innate lymphocytes that produce cytokines in response to viral infection and inflammation (64). Group 1 ILCs are comprised of noncytotoxic ILC1s and cytotoxic NK cells (65). ILC1s produce IFN-γ in response to IL-12, IL-15, and IL-18, acting as a first line of defense against viral infections (66). Given IFN-γ role as a signature pro-inflammatory cytokine, ILC1s likely stimulate inflammation in response to HSV-1 infection (67). NK cells are a type of white blood cell that can kill their targets autonomously, recognizing and eliminating cells infected with viruses or tumors (68–70). Their recruitment is mediated by chemokines such as CXCL9, CXCL10, and CCL5, which are secreted by infected corneal cells and resident DCs (53). NK cells expressing CD16 can kill HSV-infected cells opsonized with HSV-specific IgG (71). A study has been done that shows that invariant natural killer T (iNKT) cells help protect against HSV-1 because asymptomatic mice had high levels of iNKT1 cells while symptomatic mice had no iNKT cells (72). On the other hand, other studies have shown that NK cells greatly contribute to corneal damage because researchers chemically depleted NK cells in some mice, leading to the severity and frequency of HSK dropping significantly (73). Interestingly, NK cell activity is reduced even though the number of NK cells stays the same in HSK patients, meaning that the impaired function of NK cells might allow HSV-1 to reactivate more easily (74).

### 4.4 Neutrophils

Neutrophils are another type of white blood cell and act as the first line of defense by engulfing and digesting microorganisms while also releasing enzymes and toxins to kill pathogens and promote inflammation (75). Neutrophils are recruited the earliest either by chemokines such as CXCL1, CXCL2, and CCL3 or by TLR2-myeloid differentiation primary response 88 (MyD88) signaling, which is when HSV-1 glycoproteins via TLR2 induce neutrophil-recruiting chemokines (76). Through phagocytosis, degranulation, and the release of antiviral cytokines and neutrophil extracellular traps (NETs), neutrophils help limit viral spread during the acute phase of infection (77). Neutrophils also produce cytokines and extracellular matrix-degrading proteases, which cause inflammation and tissue destruction, often leading to blindness (18). Most of the damage is done through neutrophil infiltration and neovascularization since neutrophils release cytokines and chemokines, which are proinflammatory agents; however, there are some cytokines and chemokines that are anti-inflammatory agents, which could be further studied and used in future therapies (78).

### 4.5 Mast cells

MCs function as effector, initiator, and regulator cells in innate immune responses, acting as important sentinels against infection by releasing a diverse array of inflammatory molecules such as cytokines and chemokines (79). MCs typically operate through TLR signaling, using TLR3, TLR7, and TLR9 induced activation to initiate production of an inflammatory response to virus related PAMPs (80). A previous study has shown evidence of MCs contribution to protection against HSV-2, using a “MC knock-in” mouse model to show increased production of TNF-α and IL-6 following skin infection by HSV-2 (81). However, the contribution of MCs to both ocular infection and infection by HSV-1 still requires further evidence to establish a potential relationship between MCs and HSK.

## 5 Role of sensors in DNA sensing signaling pathway in HSK

Innate immunity is the first line of defense against DNA virus HSV-1. Activation of innate immunity usually requires the recognition of viral PAMPs, such as dsDNA from HSV-1, by PRRs on innate immune cells (19, 29, 82). However, DNA sensors can also recognize endogenous DNA released during cellular damage or stress, triggering immune responses that clear damaged cells and induce cytokines release (83).The cytoplasmic DNA sensors involved in HSV-1 detection include cGAS (20), interferon gamma-inducible protein 16 (IFI16) (84), DEAD-box helicase 41 (DDX41) (85), and absent in melanoma 2 (AIM2) (86), which recognize double-stranded DNA in the cytoplasm and trigger the production of type I IFN through stimulator of interferon genes (STING) signaling (29, 30, 87). The roles of these DNA sensors in HSV-1 recognition are discussed below.

### 5.1 cGAS

**Figure 2:**
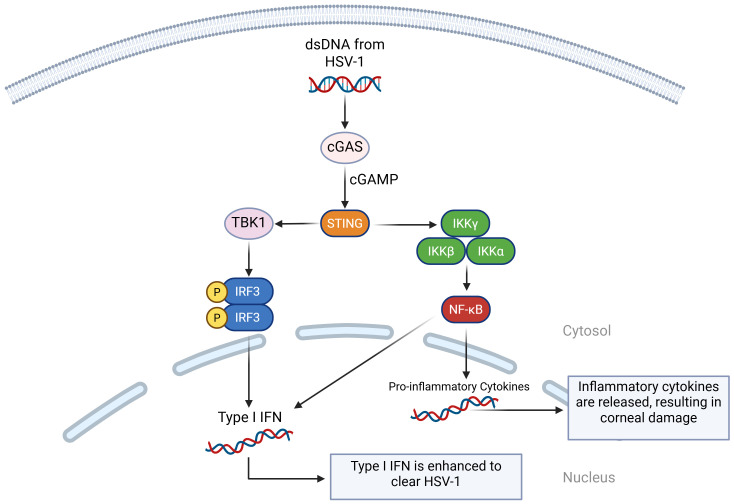
A visual diagram of the cGAS-cGAMP-STING pathway in HSV-1 infection. When cGAS detects double-stranded DNA (dsDNA) from HSV-1, it binds to it and activates enzymatic activity. This catalyzes the formation of cGAMP, which binds it to STING. STING recruits TANK-binding kinase 1 (TBK1), which phosphorylates IRF3, activating type I IFN. STING also activates IκB kinase (IKK) complex, which activates NF-κB. NF-κB translocates to the nucleus and initiates the transcription of inflammatory genes. It also works with IRF3 to initiate type I IFN.

The cGAS-STING pathway plays an important role in host antiviral immune responses and its interactions with viral immune escape mechanisms are very important for limiting HSV-1 lysis and latent infection (88, 89). When HSV-1 virus is being replicated, the cGAS enzyme senses aberrant DNA and catalyzes the cyclic cGAMP to activate STING receptors inside the cell (90, 91). Recognition of this process activates the interferon regulatory factor 3 (IRF3) and nuclear factor kappa-light-chain-enhancer of activated B cells (NF-κB) signaling pathways, which in turn promotes the secretion of type I IFN and other pro-inflammatory cytokines (92). Beta-conjugated proteins can also promote type I IFN production in the cGAS-cGAMP-STING pathway, which can better apply anti-HSV-1 effects (93) (**Figure 2**).

### 5.2 IFI16

**Figure 3:**
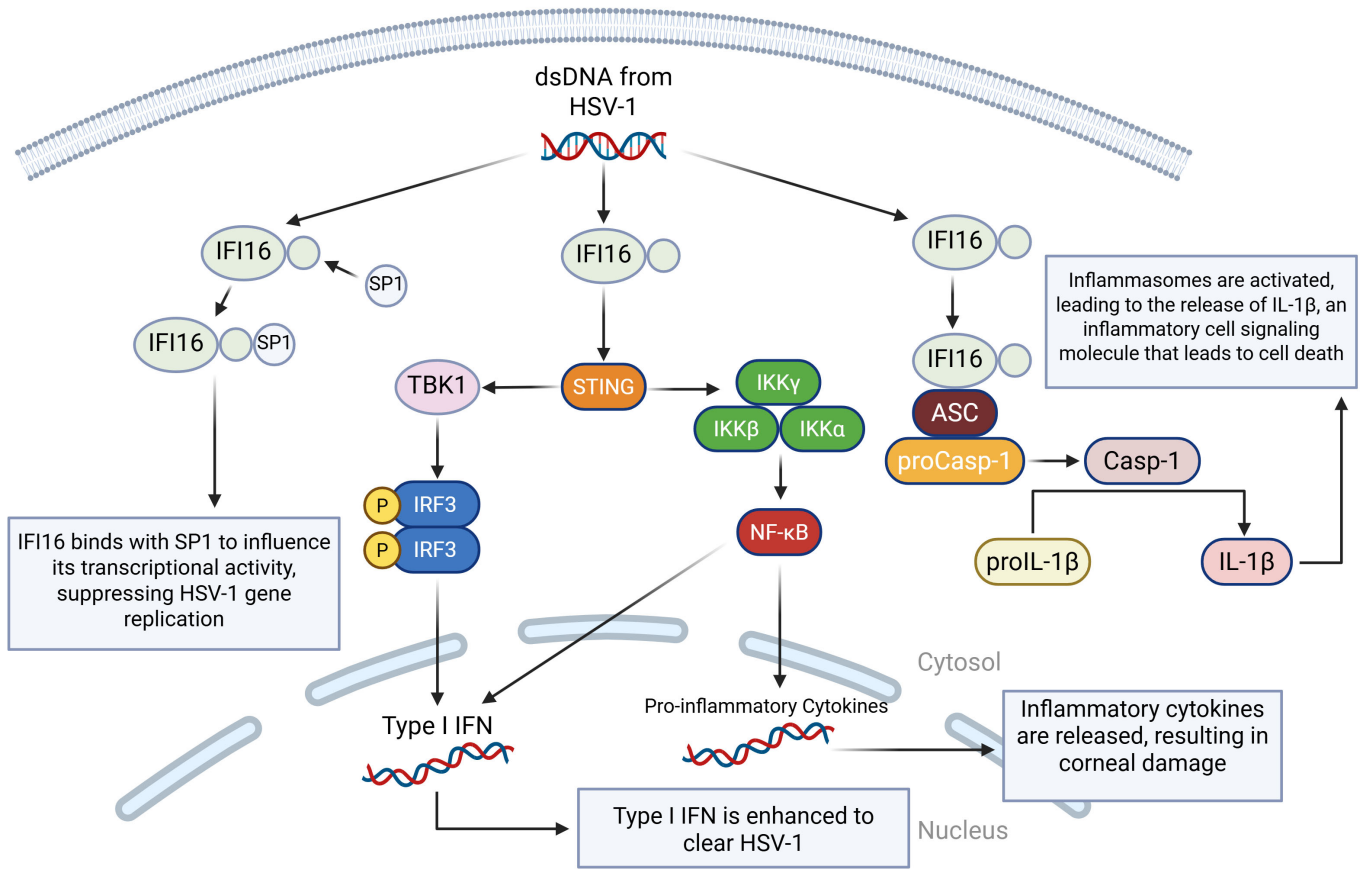
A visual diagram of the IFI16 DNA sensing pathways in HSV-1 infection. IFI16 binds to dsDNA from HSV-1. In the first pathway, IFI16 binds to the specificity protein 1 (SP1), modulating SP1’s ability to regulate antiviral genes, which ultimately leads to the restriction of HSV-1 viral replication by inducing an antiviral state. In the second pathway, the STING-TBK1-IRF3 pathway is activated, leading to type I and type III IFN production. In the third pathway, IFI16 binds to the apoptosis-associated speck-like protein (ASC), which binds to procaspase 1 (proCasp-1), facilitating the activation of Casp-1 and completing the activation of the inflammasome. Casp-1 then cleaves pro-interleukin-1 beta (proIL-1β) so that it can become mature IL-1β, which is a highly inflammatory cell signaling molecule that leads to pyroptotic cell death.

IFI16 has an important role in antiviral defense by activating the canonical STING/TANK binding kinase 1 (TBK1)/IRF3 signaling pathway in response to viral infections (87). During the HSV-1 infection, IFI16 recognizes and binds dsDNA in the nucleus, blocking the virus’s ability to turn its genes into proteins (94). This then leads to the production of interferons and many antiviral proteins, such as mucosal viral resistance (MxA, a GTPase), 2′-5′-oligoadenylate synthetase (OAS), and ribonuclease L (RNase L) (95). All of this stops the virus from spreading. Additionally, if IFI16 is absent, then expression of type I IFN and type III IFN is significantly reduced (96). Ubiquitin-specific peptidase 12 (USP12) promotes antiviral responses by removing ubiquitin molecules from proteins and stabilizing IFI16 (97). Like the duality of TLR2/TLR9, cGAS and IFI16 can co-recognize HSV-1 and stimulate the IRF3 pathway while also restricting viral replication by binding to viral genomes and activating inflammasomes, which are an essential part of the immune system response (98) (**Figure 3**).

### 5.3 DDX41

**Figure 4:**
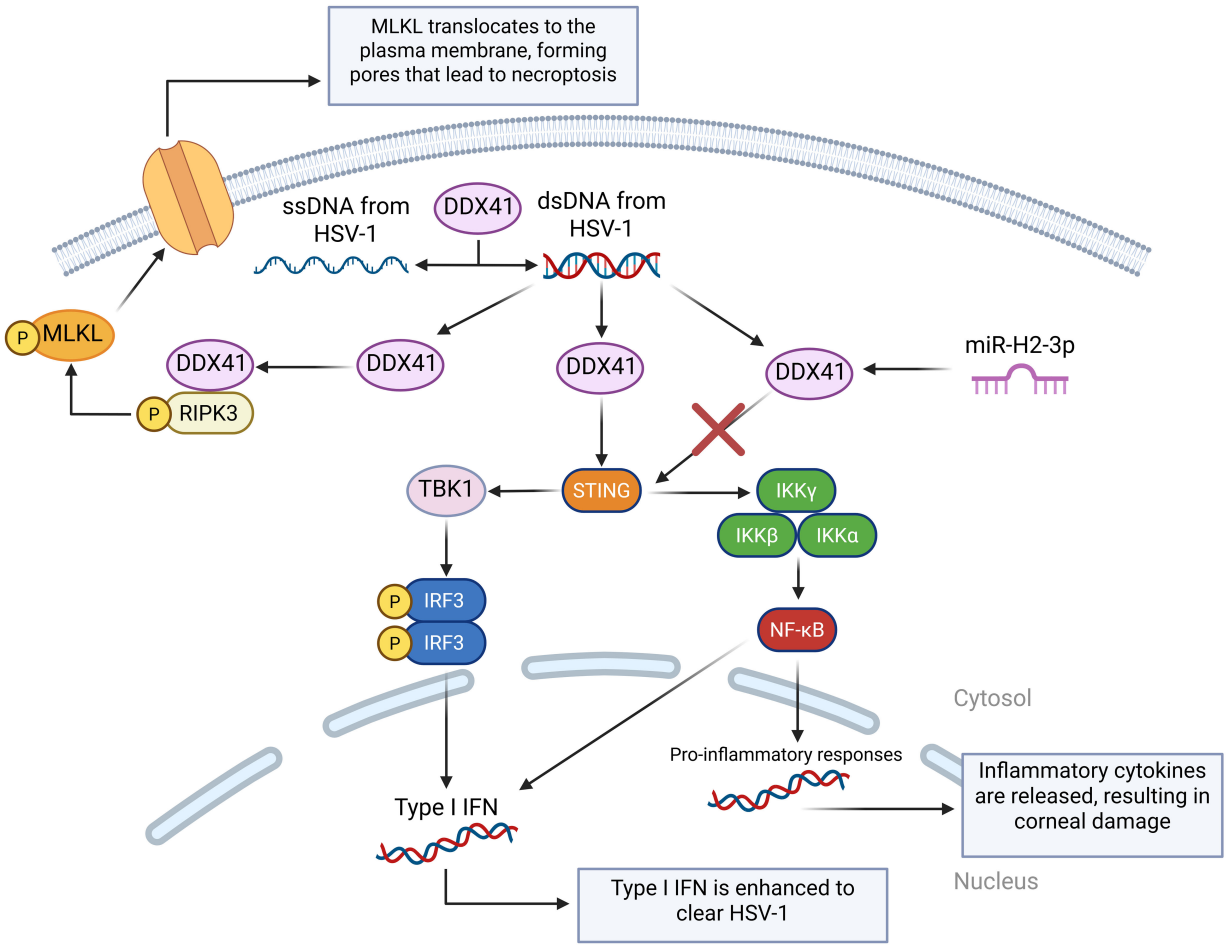
A visual diagram of the DDX41 DNA sensing pathways in HSV-1 infection. DDX41 modulates the state of cytosolic DNA by unwinding dsDNA and annealing ssDNA. This is very important in regulating cGAS activation. Like cGAS, DDX41 can also activate the STING-TBK-IRF3 pathway. miR-H2-3p targets DDX41, preventing it from activating the STING pathway and reducing the cell’s immune response. DDX41 also binds with Receptor-Interacting Serine/Threonine-Protein Kinase 3 (RIPK3) to activate the Mixed Lineage Kinase Domain-Like protein (MLKL), which translocates to the cell membrane and forms pores, disrupting the cell’s ion balance to cause necroptosis.

DDX41 is an intracellular DNA sensor that triggers the downstream pathway, requiring the adaptor STING, the kinase TBK1, and the transcription factor IRF3 to activate the type I IFN response (85), and it also plays an important role in modulating dsDNA and ssDNA from HSV-1 while also activating the DDX41– Receptor-interacting protein kinase 3 – Mixed lineage kinase domain-like protein (DDX41-RIPK3-MLKL), which results in necroptosis (99). A study was performed to screen, identify, and characterize HSV-1-encoded microRNA H2-3p (miR-H2-3p) as a suppressor of the cytosolic DNA-stimulated antiviral innate immune pathway by targeting DNA sensor DDX41 to neutralize the production of type I IFN and strengthen HSV-1 immune evasion (100) (**Figure 4**).

### 5.4 AIM2

**Figure 5:**
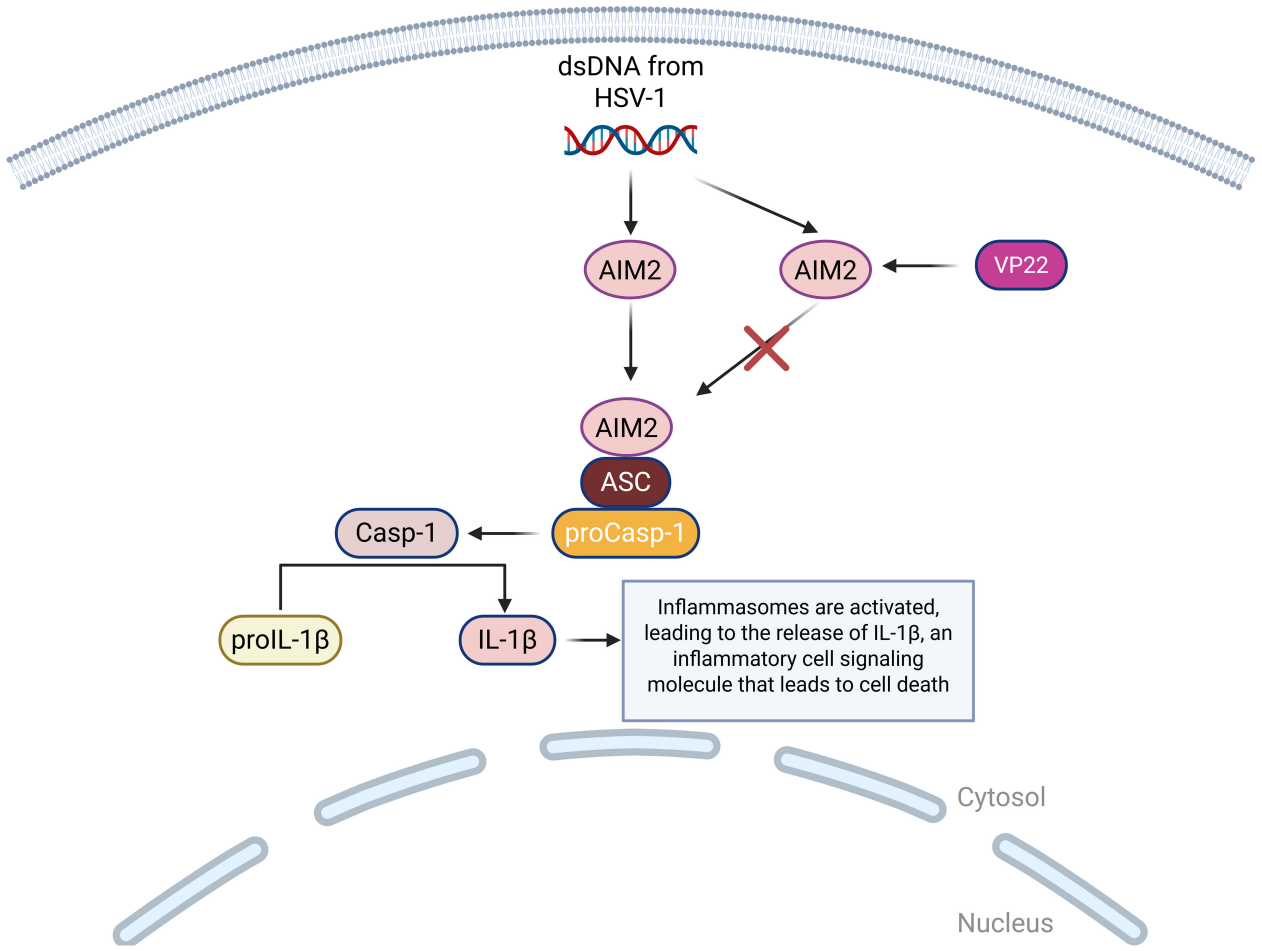
A visual diagram of the AIM2 DNA sensing pathways in HSV-1 infection. AIM2 binds to ASC, which binds to proCasp-1, facilitating the activation of Casp-1 and completing the activation of the inflammasome. Casp-1 then cleaves proIL-1β so that it can become mature IL-1β, which is a highly inflammatory cell signaling molecule that leads to pyroptotic cell death. This pathway can be inhibited by HSV-1 encoded protein VP22, which suppresses the AIM2 inflammasome activation.

AIM2 is a DNA sensor that detects foreign dsDNA in the cytoplasm, which comes from viruses like cytomegalovirus (CMV) and HSV-1 (101). When AIM2 detects dsDNA from HSV-1, it assembles an inflammasome, which is a multi-protein complex that forms inside the cell as part of the innate immune system (102). The role of inflammasomes is to detect dangerous signals from foreign invaders and trigger proptosis, a form of programmed cell death (103). The activation of the AIM2 inflammasome is triggered by dsDNA, which then results in the activation of caspase-1 and the release of pro-inflammatory cytokines IL-1β and IL-18, which play an important role in the inflammatory response of cells (86). Investigation into the AIM2 inflammasome unveiled that HSV-1 triggered the activation of AIM2 in macrophages independently of the dsDNA sensor, which means that HSV-1 can activate AIM2 without relying on the usual DNA-sensing mechanism (104). HSV-1 tegument protein VP22 (VP22), was identified as a specific inhibitor of the AIM2 inflammasome during HSV-1 infection, meaning that HSV-1 tries to block AIM2 using the protein VP22 to inhibit detection (105) (**Figure 5**).

## 6 Role of regulators in DNA sensing signaling pathway in HSK

### 6.1 TRIM family proteins

TRIM proteins, including 80 members in humans, are E3 ubiquitin ligases and play extremely important roles in regulating innate immune sensing, interferon production, and antiviral restriction (106, 107). TRIM21 can play a significant role in HSK (108). TRIM21 regulates the type I IFN response to viruses (109), and also serves as a cytosolic Fc receptor for immunoglobulin (110). HSV-1 is sensitive to type I IFN and neutralizing antibody, and the role of TRIM21 in the response to ocular HSV-1 infection in mice has been investigated (111). It has been shown that the absence of TRIM21 results in a significant increase in HSV-1 titers recovered from the thapsigargin (TG) of TRIM21 KO mice during HSV-1 infection (112). In epithelial HSK mice models, the expression TRIM21 was detected, and the clinical relationship was then investigated between TRIM21 and epithelial HSK in which TRIM21 was silenced, significantly controlling viral particle release at 1, 3, and 5 days post-HSV-1 infection (113). Ultimately, clinical scores and histopathology examinations have shown that TRIM21 can successfully reduce the severity of epithelial HSK (114).

TRIM29 has been shown to play important roles in host defense against both DNA and RNA viruses through regulating host innate immune responses mediated by type I IFN, IFN-γ, and inflammasomes (49, 69, 115–118). Specifically, TRIM29 interacts with STING to induce K48-linked ubiquitination and degradation of STING, thereby reducing type I IFN production in DCs, leading to increased HSV-1 replication and pathogenesis *in vivo* (116). Our unpublished data shows that TRIM29 is highly expressed in CECs, suggesting that TRIM29 plays a key role in controlling HSV-1 infection and may influence the severity of HSK.

TRIM18 is an E3 ubiquitin ligase that plays a negative regulatory role in the innate immune response to both DNA and RNA viruses. TRIM18 is shown to recruit protein phosphatase 1A (PPM1A) to dephosphorylate TBK1, which deactivates TBK1 to block TBK1 from interacting with its upstream adaptor STING in macrophages, thereby dampening type I IFN-mediated antiviral signaling during HSV-1 infection (62). Given that the critical role of macrophages in regulating HSK, we hypothesize that TRIM18 could regulate antiviral innate immunity in macrophages to control HSK.

### 6.2 TLR2

TLR2 is shown to detect viral glycoproteins, including HSV-1 glycoproteins gB and gH, signaling through MyD88 to activate NF-κB and mitogen-activated protein kinase (MAPK) pathways, leading to pro-inflammatory cytokine production (76). While not a DNA sensor itself, TLR2 can indirectly regulate DNA sensing pathways, such as cGAS-STING, through inflammatory priming and signaling crosstalk (119). For example, TLR2-induced cytokines like IL-1β can enhance STING pathway activation, thereby regulating type I IFN production downstream of DNA sensors (120). In murine models, TLR2 is critical for early innate responses in the cornea, with TLR2-deficient mice showing lower early inflammatory cytokine levels, reduced recruitment of neutrophils and monocytes, and decreased severity of corneal immunopathology (121). Therefore, while TLR2 helps detect HSV early, excessive TLR2 signaling drives corneal opacity, neovascularization, and scarring, ultimately damaging the cornea and causing the progression of HSK (122).

### 6.3 NLRP3

NLRP3 is a cytosolic PRR that forms the NLRP3 inflammasome, and upon activation, NLRP3 recruits apoptosis-associated speck-like protein containing a CARD (ASC) and caspase-1, driving inflammation (123). HSV-1 infection triggers NLRP3 inflammasome activation in corneal epithelial cells, stromal keratocytes, and infiltrating leukocytes (124). NLRP3-deficient mice infected with HSV-1 show reduced IL-1β secretion, lower neutrophil infiltration, and less corneal opacity and neovascularization. However, viral titers can remain similar, indicating that NLRP3 mainly drives immunopathology rather than clearance (125).

## 7 HSV-1 evasion of host innate immunity

Although the cytosolic DNA sensing signaling pathway is activated during viral infection, HSV-1 has developed multiple mechanisms to evade host antiviral innate immunity and to facilitate viral infection and replication (22, 89, 126). HSV-1 encoded proteins US11 (127), US3 (128), UL36 (129), and VP16 (130) can evade RNA sensing antiviral signaling pathways, while UL41 (131), VP24 (132), ICP0 (133), and ICP27 (134) proteins evade DNA sensing antiviral signaling pathways. Additionally, the tegument protein VP22 inhibits AIM2-dependent inflammasome responses (102). HSV-1 can also block autophagy in order to evade innate immunity. It accomplishes this through ICP34.5, which binds to Beclin-1, a key autophagy protein (135). Finally, HSV-1 can interfere with NK cells activation signals by downregulating ligands that bind to the NK-cell activating receptor NKG2D, which limits NK cells recognition and cytotoxic killing (136).

## 8 Therapeutic treatments for HSK

### 8.1 Antiviral drug therapies

Antiviral drugs, including acyclovir, trifluridine, ganciclovir, vidarabine, and famciclovir (137), are commonly used to treat HSK. Oral acyclovir, when added to primary treatment with topical corticosteroids and trifluridine, does not significantly improve initial outcomes but may provide long-term vision benefits (138). Trifluridine, a nucleoside analog, inhibits viral DNA synthesis, preventing HSV-1 replication. Higher doses have been shown to reduce the risk of antiviral resistance (139). In epithelial HSK, topical trifluridine or ganciclovir is standard, with optional oral acyclovir. For stromal and endothelial HSK, oral acyclovir is combined with topical corticosteroids (140).

### 8.2 Host-directed therapies

Host-directed therapies (HDTs) enhance host immune responses by targeting host factors critical for viral pathogenesis, offering a promising alternative to conventional antivirals for HSK (141). Unlike traditional antivirals, HDTs focus on host pathways to disrupt viral entry, replication, or immune evasion, potentially overcoming resistance to drugs like acyclovir. HSV-1 entry begins with glycoproteins binding to host cell receptors, such as heparan sulfate proteoglycans (HSPGs), followed by interactions with key glycoprotein D (gD) receptors: herpesvirus entry mediator (HVEM), nectin-1, and 3-O-sulfated heparan sulfate (3-OS HS). These interactions activate gB, facilitating membrane fusion and viral entry. HDTs can block or modify these attachment sites, particularly 3-OS HS, or use inhibitors, antagonists, or decoy molecules to disrupt HVEM or nectin-1 binding, preventing viral entry (142). HSV-1 infection also upregulates host kinases, which serve as viable therapeutic targets. For instance, the cyclin-dependent kinase (CDK) inhibitor FIT-039 disrupts mRNA transcription, inhibiting replication of various DNA viruses, including HSV-1, as shown in animal models (143). Similarly, BX795 hydrochloride, a serine/threonine kinase inhibitor, blocks viral protein synthesis, demonstrating efficacy against acyclovir-resistant HSV-1 strains in a mouse HSK model (144). Additionally, HDTs can amplify antiviral immunity by targeting immune cells. For example, overexpression of NLRP12 enhances macrophage immune responses to alleviate HSK (63), and targeted delivery of HSV-1 gD to CD169^+^ macrophages using ganglioside liposomes reduces HSK severity in mice (10). DCs are also critical targets, as local cDCs depletion results in decreased corneal nerve infection and mortality of mice (145), while pDCs depletion leads to severe HSK (37). Modulating DCs function could thus enhance antiviral defenses and mitigate HSK progression (146). Overall, HDTs offer a multifaceted approach to HSK treatment by targeting viral entry, host kinases, and immune cell responses, providing potential solutions for drug-resistant strains and improving therapeutic outcomes.

### 8.3 Anti-inflammatory therapies

Corticosteroids are anti-inflammatory and immunosuppressive drugs that decrease the production of inflammatory cytokines by binding to glucocorticoid receptors inside cells, and they are important in treating HSK because they can prevent inflammatory complications, slowing down vision impairment (147). Some corticosteroids include prednisolone acetate and dexamethasone, and they are always used with antivirals to prevent uncontrolled HSV-1 replication (148). Another type of anti-inflammatory therapy is non-steroidal anti-inflammatory drugs (NSAIDs), which are a type of medication that block cyclooxygenase enzymes in order to reduce inflammation (137). Topical NSAIDs, such as flurbiprofen and diclofenac, inhibit prostaglandin synthesis, thereby decreasing vasodilation, vascular permeability, and pain signaling (149).

### 8.4 Novel gene therapies

One of the most promising novel gene therapies is HSV-1-erasing lentiviral particles (HELP). This gene therapy uses virus-like particles to deliver SpCas9 mRNA and single-guide RNAs (sgRNAs) targeting HSV-1 genes UL8 and UL29 via corneal intrastromal injection. Preclinical studies demonstrate complete inhibition of HSV-1 replication and prevention of HSK in multiple animal models (2). Another gene therapy is meganuclease-based gene editing, which uses a meganuclease to target HSV-1 UL19, and it is delivered via adeno-associated virus serotype 2 (AAV2) to corneal grafts. In rabbit models, treated corneal transplants resisted HSV-1 infection, preventing opacity and edema (150). Host gene targeting is another type of gene therapy that uses CRISPR to edit NECTIN-1, an essential HSV-1 entry receptor on CECs. Studies have shown that lentiviral delivery *in vitro* dramatically lowered infection rates and viral load (151).

### 8.5 Combination therapies

**Figure 6:**
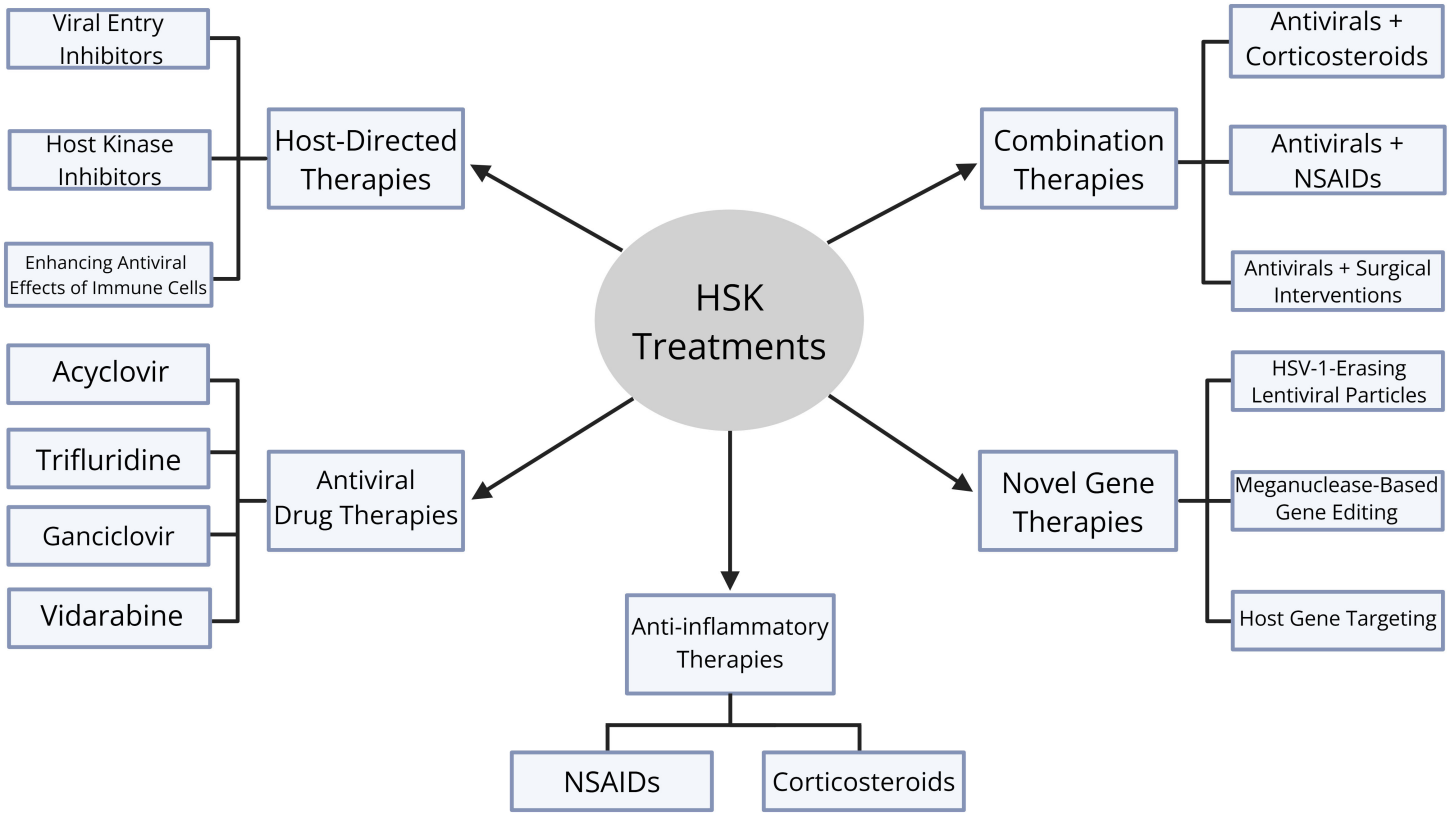
Diagram of therapeutic treatments for HSK. There are five categories of therapeutic treatments: antiviral drug therapies, host-directed therapies, anti-inflammatory therapies, novel gene therapies, and combination therapies. Antiviral drug therapies include acyclovir, trifluridine, ganciclovir, and vidarbine. Host-directed therapies include viral entry inhibitors, host kinase inhibitors, and enhancing antiviral effects of immune cells. Anti-inflammatory therapies include corticosteroids and non-steroidal anti-inflammatory drugs (NSAIDs). Novel gene therapies include HSV-1-erasing lentiviral particles, meganuclease-based gene editing, and host gene targeting. Finally, combination therapies include antivirals and corticosteroids, antivirals and NSAIDs, and antivirals and surgical interventions.

Combination therapies address both viral replication and immune-mediated corneal damage in HSK management. The most common type of combination therapy is antivirals and corticosteroids. This is especially effective in treating stromal and endothelial HSK, and an example would be oral acyclovir combined with topical prednisolone acetate (152). Another type of combination therapy is antivirals and NSAIDS, which is typically used when steroids are contraindicated. An example is topical trifluridine and topical flurbiprofen, but the downside to this combination treatment is that NSAIDs are less effective than corticosteroids for stromal inflammation (153). Finally, antivirals can be combined with surgical interventions, which is a treatment method that is used in severe recurrent HSK with scarring. An example is oral acyclovir prophylaxis and penetrating keratoplasty, which is a treatment plan that has been shown to reduce the risk of HSK recurrence and graft failure (154) (**Figure 6**).

## 9 Conclusions and future perspectives

HSK is a complex disease that involves many different aspects, including the viral infection itself, the immune system’s reaction, molecular regulation, and inflammation. HSK is the most common cause of infectious blindness, and the current methods of treatment are unsatisfactory. As a dsDNA virus that exhibits a strong neurotropic nature, treatment can be extremely difficult since it persists in neuronal tissues and can exist in a latent phase while preventing host immune clearance. With many different DNA sensing pathways, the immune system’s response to HSV-1 is extremely complex and involves many interconnected interactions between various immune cells. Novel insights into disease immunopathogenesis could allow for the development of more efficient and effective therapeutic options. Current therapies, while effective at controlling viral replication, are limited in preventing corneal scarring, opacity, and neovascularization. Because of this, increasing attention has turned toward host-directed therapies that modulate innate immune responses. Some potential targets for future host-directed therapies include cGAS-STING, NLRP3, IL-17, and TRIM proteins. Looking forward, integrating antiviral agents with precision immunomodulation offers a path forward for more effective and personalized HSK management. Future research should prioritize clinical translation of host-targeted interventions as well as combination strategies that balance viral control with immune regulation.
